# Plasma activated Ezhangfeng Cuji as innovative antifungal agent and its inactivation mechanism

**DOI:** 10.1186/s13568-023-01571-6

**Published:** 2023-06-27

**Authors:** Lin Lin, Yue Zhuo, Qiran Dong, Chunjun Yang, Cheng Cheng, Taofeng Liu

**Affiliations:** 1 The Postgraduate School of Anhui, University of Chinese Medicine, Hefei, 230012 People’s Republic of China; 2 Department of Dermatology, The First Affiliated Hospital of Anhui University of Chinese Medicine, Hefei, 230012 People’s Republic of China; 3 Nanjing University of Chinese Medicine, Nanjing, 210023 People’s Republic of China; 4 Department of Dermatology, The Second Affiliated Hospital, Anhui Medical University, Hefei, 230601 People’s Republic of China; 5 Institute of Plasma Physics, Chinese Academy of Sciences, Hefei, 230031 People’s Republic of China

**Keywords:** Plasma activated Ezhangfeng Cuji, *Candida albicans*, In vitro antifungal effect, Fungal structure, Virulence factor

## Abstract

*Candida albicans* is a highly drug-resistant fungus for which new treatments are urgently needed due to the lack of clinically effective options. In this study, we evaluated the antifungal activity and mechanism of plasma-activated Ezhangfeng Cuji (PAEC) against *Candida albicans* and compared it with physiological saline (PS), plasma-activated physiological saline (PAPS) and Ezhangfeng Cuji (EC). After dielectric barrier discharge (DBD) plasma treatment with EC for 20 min followed by a 10 min immersion of *Candida albicans*, the fungus was reduced by approximately 3 orders of magnitude. High performance liquid chromatography (HPLC) results showed an increase of 41.18% and 129.88% in the concentration of oxymatrine and rhein, respectively, after plasma-treated EC. The concentrations of reactive species (RS), such as H_2_O_2_, ${\text{NO}}_{3}^{ - }$, and O_3_, were found to be higher and the pH value was getting lower in PS after plasma treatment. Detailed analysis of intracellular material leakage, reactive oxygen species (ROS), apoptosis for *Candida albicans* and observation by transmission electron microscopy (TEM) and scanning electron microscopy (SEM) demonstrated that PAPS, EC and PAEC disrupt the morphological structure of *Candida albicans* to varying degrees.Additionally, specific analyses on *Candida albicans* virulence factors, such as adhesion to tissue surfaces, cell surface hydrophobicity (CSH), the transition of yeast-phase cells to mycelium-phase cells, and the secretion of hydrolytic enzymes for *Candida albicans* were conducted and found to be inhibited after PAPS/EC/PAEC treatment. In our investigation, the inhibitory effects on *Candida albicans* were ranked from strong to weak as follows: PAEC, EC, PAPS, and PS.

## Key Points

1. PAEC inactivates the fungus more effectively than PAPS and EC.
2. PAEC can destroy fungal morphological structure.
3. PAEC can reduce fungal virulence factors.
4. PAEC’s antifungal ability is due to the synergy between RS and TCM components.

## Introduction

*Candida albicans* is a common pathogenic fungus and the leading cause of severe fungal infections and nosocomial infections in hospitals (Gow et al. 2017). Clinically, it can result in thrush, colpitis mycotic, diaper rash, and even blood-borne disseminated infections on the mucosa and skin (Nobile et al. 2015). The three primary groups of antifungal medications currently on the market are polyenes, azoles, and echinocandins (De Cremer et al. 2015). At present, only two specific targets of action exist for antifungal medications against *Candida*: azoles and polyenes act on ergosterol, a component of the cell membrane, while echinocandins operate on β-1, 3-glucan, a component of the cell wall (Whaley et al. 2016). Due to antifungal drug usage in recent years, drug resistance in *Candida* has gradually increased and is prone to recurrence, which seriously affects physical and mental health. In addition to skin disease, *Candida albicans* can cause systemic infections. The mortality rate of patients suffering from *Candida albicans* has been increasing every year in recent years due to drug resistance (Lee et al. 2021).

The pathogenic ability of *Candida albicans* is primarily determined by the pathogenic invasion route, site, and number, as well as the cytotoxicity and immunity of the organism. The transition from commensal to the pathogenic organism is attributed to the selective expression of large amounts of virulence factors under appropriate susceptibility conditions (Mayer et al. 2013). *Candida* virulence factors are abundantly expressed, allowing *Candida* to invade host cells and cause deep fungal infections in various sites and organs (Gow 2013). The elements and processes that influence the ability of *Candida* to spread infection or cause disease are referred to as its "fungal virulence". The main *Candida* virulence factors include adhesion to tissue surfaces, cell surface hydrophobicity (Menezes et al. 2013), the transformation of yeast-phase cells to mycelium-phase cells, secretion of hydrolytic enzymes (Santos et al. 2013), and biofilm formation (Gulati et al. 2016). Fungal virulence factors also include the ability to adapt to the environment and the production of mutations in response to external stressors (Berman et al. 2002). Researchers have increasingly realized that limiting the production of virulence factors in *Candida albicans*, thereby suppressing its ability to cause infections by reducing its potential to cause disease, offers an alternative therapy for conditionally harmful fungus to standard antifungal medications (Vila et al. 2017).

External treatment is commonly used in the clinical treatment of superficial *Candidiasis* in traditional Chinese medicine (TCM) due to its safety and cost-effectiveness. Many plant-derived molecules, such as those isolated from herbal medicines, have been shown to inhibit fungal activity via various targets such as cell membranes, cell walls, mitochondria, and virulence factors (Liu et al. 2017). For example, Berberine hydrochloride, a TCM component, can inhibit biofilm formation by inhibiting the cellular activity of early biofilms, disrupting the microscopic morphology of *Candida albicans*, and reducing biofilm thickness (Huang et al. 2020). Another example is that the ability of *C. longum's* main active ingredient, loureirin A, to inhibit fungal biofilms may be related to the inhibition of pathogenic traits, adhesion, and mycelium formation (Lin et al. 2019). Chinese medicine requires lengthy exterior washing; it must soak for 30 min each day for at least a month. The use of TCM alone will gradually lessen the antifungal impact and make it easy for the condition to recur because *Candida* is prone to antibiotic resistance. It is necessary to find a new alternative technology that complements Western medicine or physics to improve the antifungal effect of external Chinese medicine and streamline the application stages.

Plasma, the fourth state of matter, can be categorized into high-temperature and low-temperature plasma. The potential clinical applications of low-temperature plasma in treating dermatological conditions and other diseases have recently garnered significant attention. While numerous studies have demonstrated the effective inactivation of bacteria by plasma, few studies have been conducted on fungi and have found antifungal effects to be insignificant (Niedźwiedź et al. 2019; Casas-Junco et al. 2019). According to Nishime et al. (2017) the cause may be that fungi are eukaryotic cells with very thick cell walls made of tough polysaccharide layers, like chitin, which give the wall its structural strength and increase its resistance to dangerous external chemicals. Shi et al. (2008) found that plasma disrupts the external structure of *Candida albicans*, causing the release of cytoplasm and resulting in fungal death. Rahimi-Verki et al. (2016) discovered that low-temperature plasma significantly reduces the growth of *Candida albicans* in vitro by inhibiting ergosterol synthesis and decreasing key fungal virulence factors such as biofilm formation, phospholipase activity, and protease activity.

In addition to the direct inactivation of bacteria by plasma, the antimicrobial properties of plasma-activated solutions have also attracted considerable interest. Plasma-activated oil releases significant amounts of ozone and ozone oxide, which exhibit potent antibacterial effects against *E. coli* (Zou et al. 2019). Plasma-activated water has been shown to have both a short-term rapid inactivation effect on S. aureus and a time-dependent long-term inhibitory effect on *S. aureus* biofilm reproduction (Xu et al. 2020). Furthermore, plasma-activated liquid (PAL) has been found to have antibacterial effects on *Neisseria gonorrhoeae* biofilm, with long-lived RS in PAL disrupting cell membranes and altering the balance of reactive oxygen species (ROS) within cells, ultimately inhibiting bacterial metabolism and leading to bacterial death (Liu et al. 2021a). Chinese herbal medicines have a wide range of clinical applications in the treatment of skin diseases, particularly in traditional antibacterial and antifungal applications that are typically used in solution form. While previous research suggests that plasma-activated solutions may exhibit enhanced antibacterial properties, the effects of plasma-activated TCM solutions on antibacterial has activity have not yet been investigated.

In this study, we selected Ezhangfeng Cuji (EC), a dermatomycosistreatment used at the First Affiliated Hospital of Anhui University of Chinese Medicine, as a representative compound herbal antifungal medicine. EC contains *Cortex Pseudolaricis*, *Radix Stemonae*, *Rhubarb*, *Matrine*, *Phellodendron*, and *Sichuan pepper* as its main ingredients and uses vinegar (In Chinese, it is named Cuji) as a solvent to extract the active compounds. Our aim was to evaluate the antifungal impact of plasma-activated Ezhangfeng Cuji (PAEC) on *C. albicans* and to investigate its mechanism of action.

## Materials and methods

### Atmospheric pressure air plasma

**Fig. 1:**
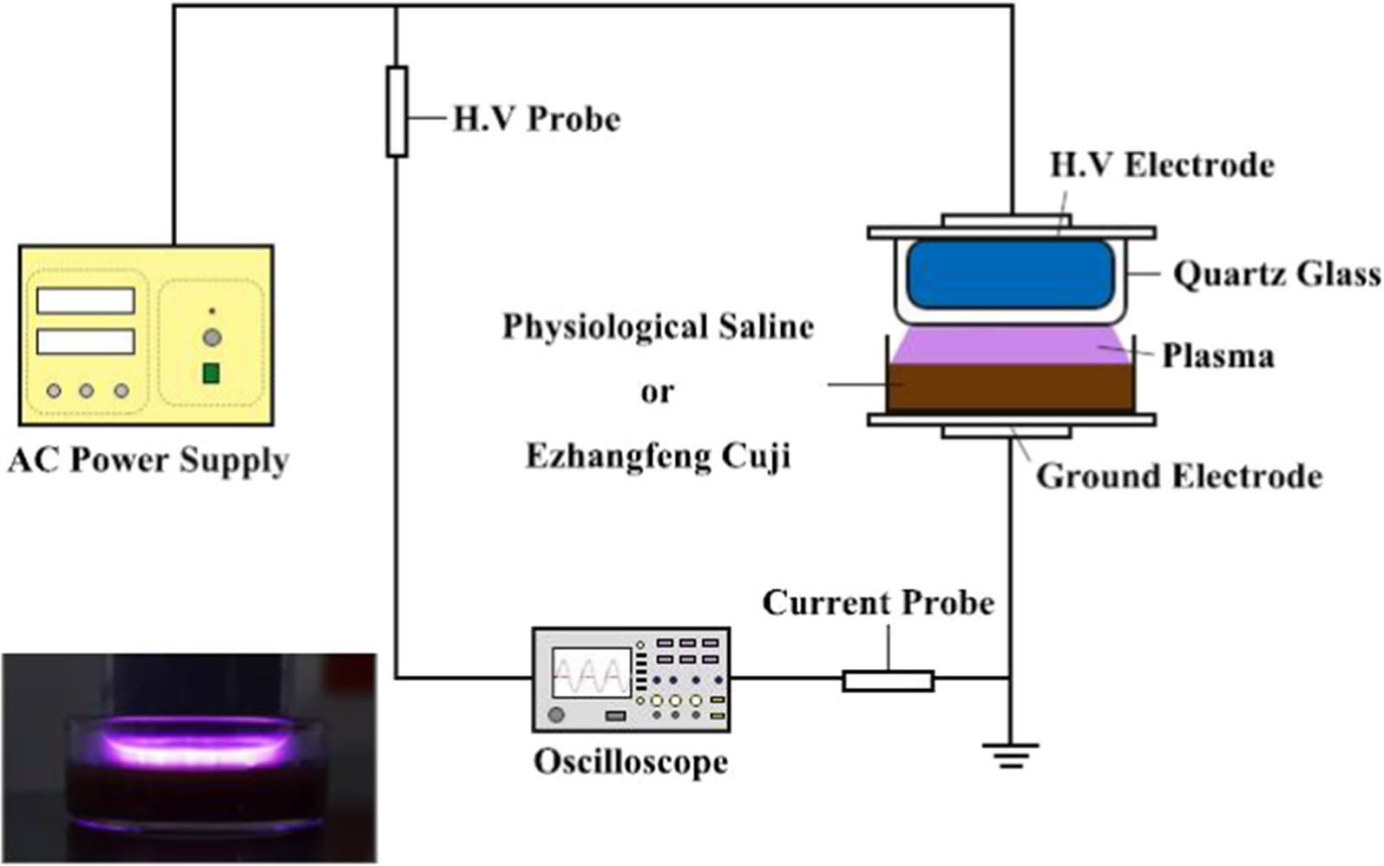
Schematic diagram of the dielectric barrier discharge (DBD) plasma source

The air DBD plasma apparatus used to prepare PAL under atmospheric pressure is shown in Fig. 1, with specific parameters described in our previous study (Xu et al. 2022). Discharge voltage and current waveforms were acquired by a Tektronix MSO5104 digital oscilloscope with a high-voltage probe (P6015A) and a current probe (P6021) (data not shown). A Petri dish containing 7 mL of PS or EC was placed on the ground electrode, with the distance between the liquid surface and the high voltage electrode set at 5 mm. All experiments were conducted with the power supply operating at a maximum voltage of 15 kV and a discharge current of approximately 100 mA. PS and EC were exposed to plasma for 20 min to prepare PAPS and PAEC, respectively.

### Fungal strains and culture conditions

A strain of *C. albicans* (ATCC MYA-2876) was obtained from the College of Integrated Chinese and Western Medicine at Anhui University of Chinese Medicine. The fungus was cultured overnight in Yeast Extract Peptone Dextrose Broth (YPD) (Hangzhou Microbial Reagent Co., Ltd., Hangzhou, China) at 37 °C with shaking at 120 rpm. Prior to each treatment, the fungal concentration was adjusted to (1–5) × 10^6^ CFU mL^−1^.

### Reactive species in PAPS and pH in PS/PAPS/EC/PAEC

The concentrations of three representative long-lived reactive species (RS), including H_2_O_2_, ${\text{NO}}_{3}^{ - }$, and O_3_, in PAL were measured using a PhotoLab 6100 (WTW, Germany), and corresponding test kits of No. 18789, 09713, and 00607 (Merck, Germany) (Shen et al. 2015). The pH values of PA/PAPS/EC/PAEC were determined using a microprocessor pH meter (PB-10, Sartorius).

### High-performance liquid chromatography (HPLC) analysis

HPLC was used to determine the concentrations of oxymatrine and rhein, two major components of EC. Oxymatrine and rhein standards (purity 98.0%) with CAS numbers 16837-52-8 and 478-4-3, respectively, were used. An Agilent HPLC-UV1260 series system (Agilent, UAS) equipped with a column compartment, automated sampler, and quaternary pump was used for analysis, along with a variable wavelength detector (VWD). The sample injection volume was set at 10 µL at room temperature (30.0 °C), and separation was performed using a C18 reversed-phase column (4.6 mm × 250 mm, 5 µm inner diameter of 5 µm; Eclipse XDB, Agilent, USA). For the detection of oxymatrine at 220 nm (Qiu et al. 2003), the mobile phase consisted of 15% acetonitrile (HPLC grade; Sigma-Aldrich, Germany) and 85% 0.1 phosphoric acid solution (HPLC grade) (Sigma-Aldrich, Germany). For the detection of rhein at 254 nm (Cheng et al. 2020), the mobile phase consisted of 85% methanol (HPLC grade; Sigma-Aldrich, Germany) and 15% 0.1 phosphoric acid solution.

### Inactivation of *C. albicans* by PS/PAPS/EC/PAEC

In each experiment, 3 mL of fungal suspension was mixed with 3 mL of PS/PAPS/EC/PAEC to form the reaction solution. Fungi were incubated in the reaction solution for 1, 5, and 10 min. The fungal suspensions were then serially diluted tenfold with sterile water to the desired concentrations. Aliquots (100 μL) of the diluted samples were spread on Sabouraud Dextrose Agar Plates (SDA; Hangzhou Microbial Reagent Co., Ltd., Hangzhou, China) and incubated for 48 h at 37 °C with 5% CO_2_. The number of *Candida albicans* colonies on each plate was counted.

### Fungal extracellular alkaline phosphatase (AKP) levels

Using an AKP kit (Jiancheng, Nanjing, China), the fungal extracellular AKP levels were measured. After submerging the fungal cells in PS/PAPS/EC/PAEC for 10 min, the supernatant was removed and mixed with the substrate solution and buffers. The mixture was then incubated for 15 min at 37 °C before adding a coloring agent. Samples were evaluated using a multifunctional enzyme marker (Varioskan Flash, Thermo, USA) at 520 nm (Hajdu et al. 2021).

### Fungal extracellular total protein (TP) concentration

Fungal extracellular total protein (TP) concentration was assessed using a TP kit (Jiancheng, Nanjing, China). After submerging the fungal cells in PS/PAPS/EC/PAEC for 10 min, the supernatant was collected and mixed with Coomassie blue staining solution. The mixture was allowed to sit for 15 min before being evaluated using a spectrophotometer with 595 nm wavelengths (Lee and Kim 2017).

### Fungal intracellular ROS concentration

Fungal intracellular ROS concentration was measured using a ROS test kit (Beyotime, China). After submerging the fungal cells in PS/PAPS/EC/PAEC for 10 min, the suspension was combined with a concentration of 10 µmol L^−1^ 2,7-dichlorodihydrofluorescein diacetate (DCFH-DA) solution and incubated for 15 min at 37 °C in the dark. Samples were examined using flow cytometry (Beckman, USA) at excitation/emission wavelengths of 488/525 nm and data were analyzed using FlowJo VX (Zhao et al. 2016).

### Analysis of fungal cell apoptosis

Cell apoptosis was measured using an Annexin V-FITC/PI apoptosis kit (Biosharp, China). After submerging the fungal cells in PS/PAPS/EC/PAEC for 10 min and 48 h, respectively, the suspension was combined with Binding Buffer and Annexin V-FITC. The mixture was incubated for 5–10 min at room temperature in the darkbefore adding PI. Samples were examined using flow cytometry, and results were analyzed using FlowJo VX (Yang et al. 2008).

### Structural changes

#### Scanning electron microscope

The morphological changes in the fungi treated by PS/PAPS/EC/PAEC were examined by scanning electron microscopy (SEM). The SEM images were acquired on the S4800 SEM (Hitachi, Japan) after gold–palladium coating (Stähli et al. 2021).

#### Transmission electron microscopy

By using transmission electron microscopy (TEM), the morphological alterations in the fungus treated with PS/PAPS/EC/PAEC were analyzed. The cells were treated in 5% glutaraldehyde for 3 h. The solution was then given 1% osmium tetroxide for 90 min. The samples were overnight immersed in epoxy resin. The sections were stained and examined by TEM (JEM-2100F, Japan) (Martínez et al. 2014).

### Virulence factors

#### Adhesion to tissue surfaces

Fungal adhesion to tissue surfaces was assessed using the cck-8 assay. Fungus suspensions were inoculated into 96-well plates for 1, 2, 3, and 4 h, respectively at 37 °C. After removing the supernatant, the fungal cells were submerged in PS/PAPS/EC/PAEC for 24 h at 37 °C. The samples were then combined with CCK-8 solution (Phygene, China) and incubated at 37 °C for 2 h in the dark before being evaluated using a multifunctional enzyme marker with 450 nm wavelengths (Hirasawa et al. 2018; Song et al. 2021).

#### Fungal cell surface hydrophobicity (CSH)

The hydrophobicity of *Candida. albicans* cell surface was assessed using water-hydrocarbon two-phase. Fungus cells were submerged in PS/PAPS/EC/PAEC In a 6-well plate and incubated at 37 °C for 24 h. After harvesting the fungus cells, the *Candida* suspensions were diluted with SDB medium until they had an absorbance value of 1 at 600 nm in a spectrophotometer. The suspensions (1.2 mL) were then combined with octane (0.3 mL) for 3 min mixed for 3 min using a vortex oscillator before being left for 15 min. After the two phases separated, the aqueous phase samples were transferred to clean test tubes. A spectrophotometer with wavelengths of 600 nm was used to evaluate the aqueous phase samples and determine the value of CSH.

$$\mathrm{CSH}=\frac{C0-C1}{C0},$$

where *C0* is the value at *OD600* for the sample not covered with octane, and *C1* is the value at *OD600* for the aqueous phase sample (Xu et al. 2019).

### The transformation of yeast-phase cells to mycelium-phase cells

A 100 μL portion of the fungal suspension was placed on Spider agar plates and incubated at 37 °C for 24 h after submerging the fungal cells in PS/PAPS/EC/PAEC for 10 min. Mycelium production in the samples was examined and documented using an optical microscope (OLYMPUS, Japan) (do Rosário et al. 2019).

### Secretion of hydrolytic enzymes

After submerging the fungal cells in PS/PAPS/EC/PAEC for 10 min, the fungal suspension was poured onto bovine serum albumin yolk and agar plates and cultured at 37 °C for 48 h. The activity levels of hydrolytic enzymes were established according to the Pa (for Sap) and Pz (for PL) index, calculated by the ratio of the diameter of the colony to the diameter of the colony plus that of the precipitation zone (Silva-Rocha et al. 2015). The higher the value, the less effective the secretion ability.

### Statistical analysis

All experiments were performed at least three times and averaged. Data were analyzed using analysis of variance (ANOVA) with GraphPad Prism 9 software. The significance level was set at a p-value of < 0.05.

## Results

### Reactive species generation and pH value changes

**Fig. 2:**
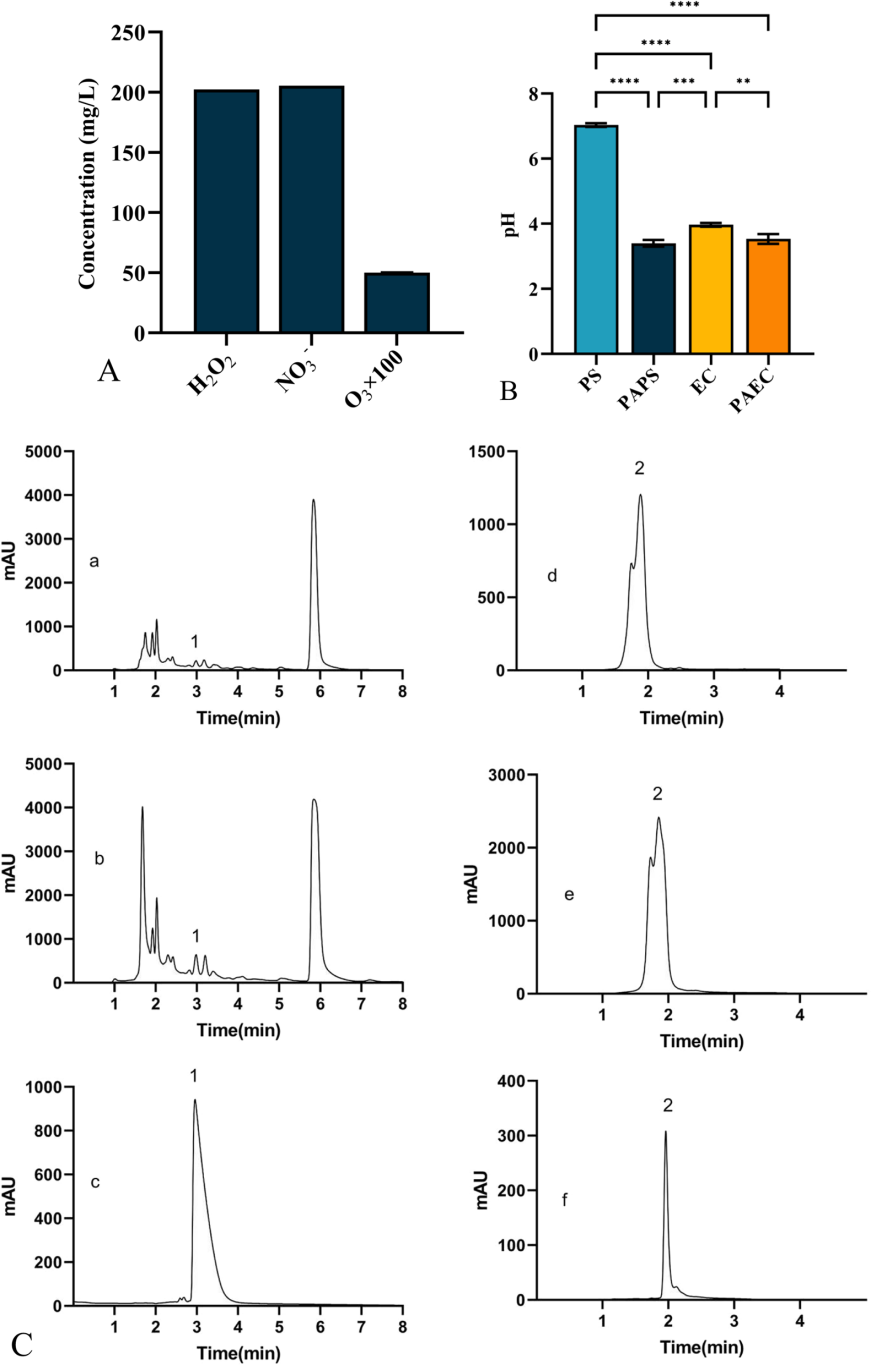
Concentrations H_2_O_2_, ${\text{NO}}_{3}^{ - }$, and O3in PAPS (**A**),the pH value of the PS, PAPS, EC, and PAEC (**B**) and HPLC chromatogram for determining the content of oxymatrine (1) and rhein (2) in EC and PAEC (**A**–**F**). **A**, **D** EC; **B**, **E**: PAEC; **C**: oxymatrine standard; **F**: rhein standard

Figure 2A shows the concentrations of long-lived aqueous RS such as H_2_O_2_, ${\text{NO}}_{3}^{ - }$, and O_3_ in PAPS. Under normal conditions, PS does not contain these substances. However, after 20 min of plasma treatment, the concentrations of H_2_O_2_, ${\text{NO}}_{3}^{ - }$, and O_3_ in PAPS increased to varying degrees. Specifically, the concentrations of H_2_O_2_ and ${\text{NO}}_{3}^{ - }$ rapidly increased to 202.4 ± 0.002 mg L^−1^ and 205.5 ± 0.05 mg L^−1^ respectively, while O_3_ levels showed a slower growing to 0.5 ± 0.008 mg L^−1^. Figure 2B displays the pH values of the PS, PAPS, EC, and PAEC. After 20 min of plasma treatment, the pH values of both PS and EC decreased, with the pH of PS decreasing from 7.03 to 3.40 and the pH of EC decreasing slightly from 3.97 to 3.53.

### Chinese herbal ingredients changes

The HPLC chromatogram in Fig. 2C examined the levels of oxymatrine and rhein, two herbal components in EC and PACE. The HPLC plots of EC, PAEC, and standard oxymatrine at a concentration of 0.5 mg L^−1^ are shown in Fig. 2Ca, Cb, and Cc respectively (with peak 1 representing oxymatrine), while Fig. 2Ce, Cf, and Cg show the HPLC plots of EC, PAEC, and standard rhein at a concentration of 0.5 mg L^−1^, respectively (with peak 2 representing rhein). After a 20-min plasma treatment, the concentrations of both oxymatrine and rhein increased to varying degrees. Using HPLC, it was found that the concentrations of oxymatrine and rhein in EC were 25.19 mg L^−1^ and 429.94 mg L^−1^, respectively. After plasma treatment, the concentration of oxymatrine in PAEC increased to 35.57 mg L^−1^ (a 41.18% increase); while the concentration of rhein rose to 988.36 mg L^−1^ (a 129.88% increase).

### Effect of plasma-activated liquid on the viability of *C. albicans*

**Fig. 3:**
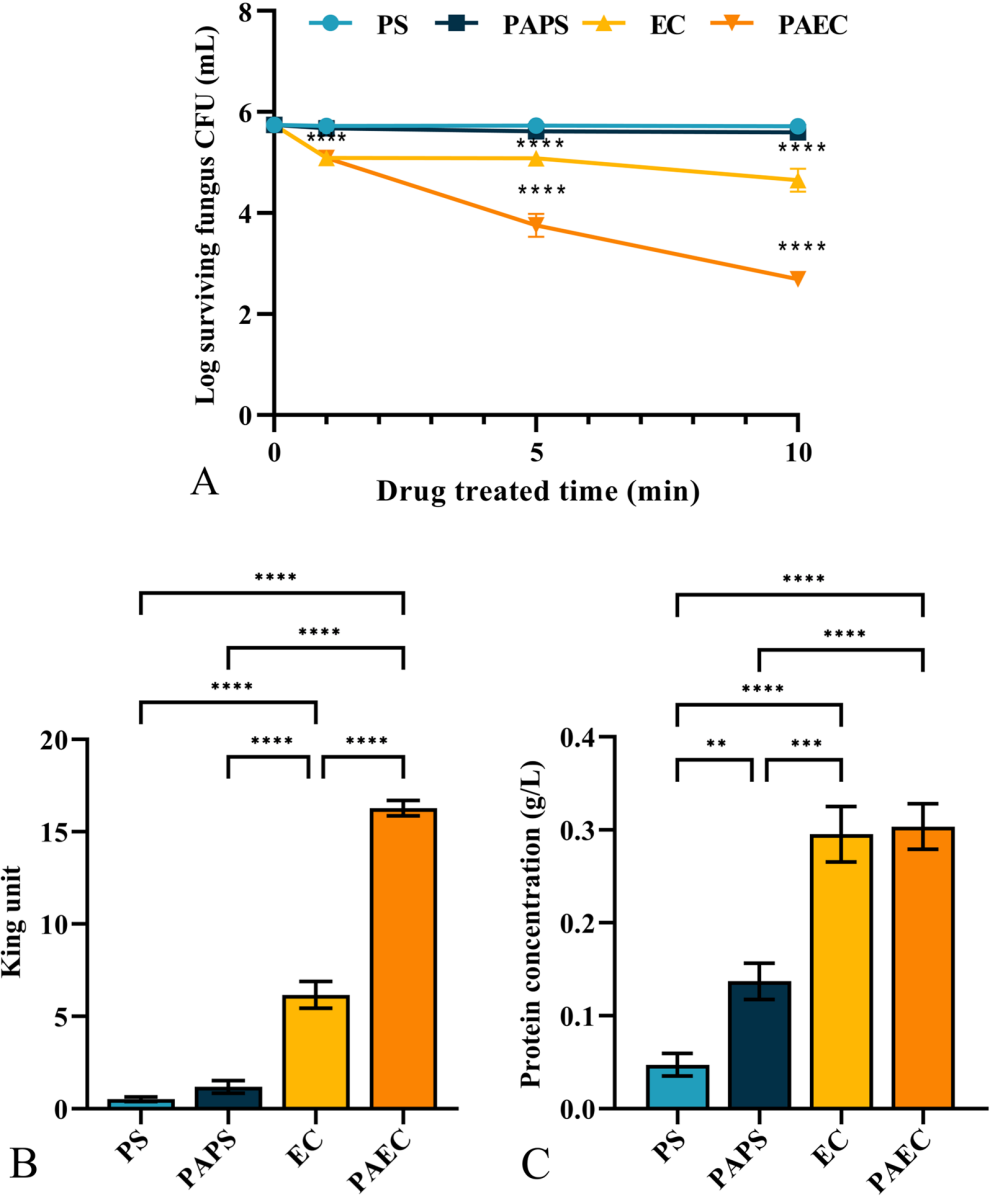
Survival curves of *Candida albicans*after treatment with the PS/PAPS/EC/PAEC under different immersion times (1, 5, 10 min, respectively) (**A**) AKP levels (**B**) and TP concentration (**B**) of fungi after treatment with the PS/PAPS/EC/PAEC

Figure 3A depicts the survival curves of *C. albicans* after immersion in PS/PAPS/EC/PAEC for 1, 5, and 10 min, with all (1–5) × 10^6^ CFU mL^−1^ of the original fungi present. As immersion time increased, the number of cultivable fungi remained essentially unchanged in PS or PAPS. However, when the EC immersion time was kept at 10 min, the number of cultivable *Candida albicans* gradually declined, resulting in a reduction of 1.089 ± 0.230 log_10_ CFU mL^−1^. In comparison, more fungi were inactivated when the PAEC immersion time was kept at 10 min, with 3.05 ± 0.027 log_10_ CFU mL^−1^ of fungi being deactivated. Overall, immersion in PS/PAPS had almost no effect on the inactivation of *Candida albicans*, while immersion in EC/PAEC significantly inhibited fungal growth, with the effect of PAEC being more pronounced.

### Permeability of fungal cell walls and cell membranes

Figure 3B displays the levels of fungal AKP after a 10 min immersion in PS/PAPS/EC/PAEC. AKP is an enzyme located between the cell wall and cell membrane. Under normal conditions, AKP is not detectable outside *Candida* cells with intact cell. The results show that there wa no significant difference between the AKP levels of fungi treated with PS and PAPS. However, the fungal AKP levels were significantly higher after immersion in EC/PAEC, with values of 6.164 ± 0.589 and 16.270 ± 0.340 King Units, respectively. This indicates that EC and PAEC had a more significant effect on the fungal AKP levels compared to PS or PAPS.

Figure 3C presents the fungal TP concentration after a 10-minuteimmersion in PS/PAPS/EC/PAEC. Normally, when the cell is intact, no macromolecules are detected outside the cell. Protein molecules can be detected outside the cell when cell rupture occurs, so protein spillage can be considered an indicator of cell rupture. The results show that compared to PS/EC, the extracellular TP content of *Candida albicans* increased to varying degrees after a 10-min immersion in PAPS/PAEC. The extracellular TP content of the fungus increased slightly after PAPS immersion compared to PS immersion, reaching 0.09 ± 0.026 g L^−1^. This indicated that PAPS was able to partially damage the cell wall and cell membrane, causing leak of AKP and TP, but had no discernible inhibitory impact on *Candida albicans* after 10 min. Meanwhile, both EC and PAEC increased the extracellular TP content with almost no difference between them. This indicates that EC and PAEC significantly damaged the cell wall and cell membrane of *Candida albicans* with PAEC having a more aggressive effect on fungal inactivation.

### Apoptosis situation changes

**Fig. 4:**
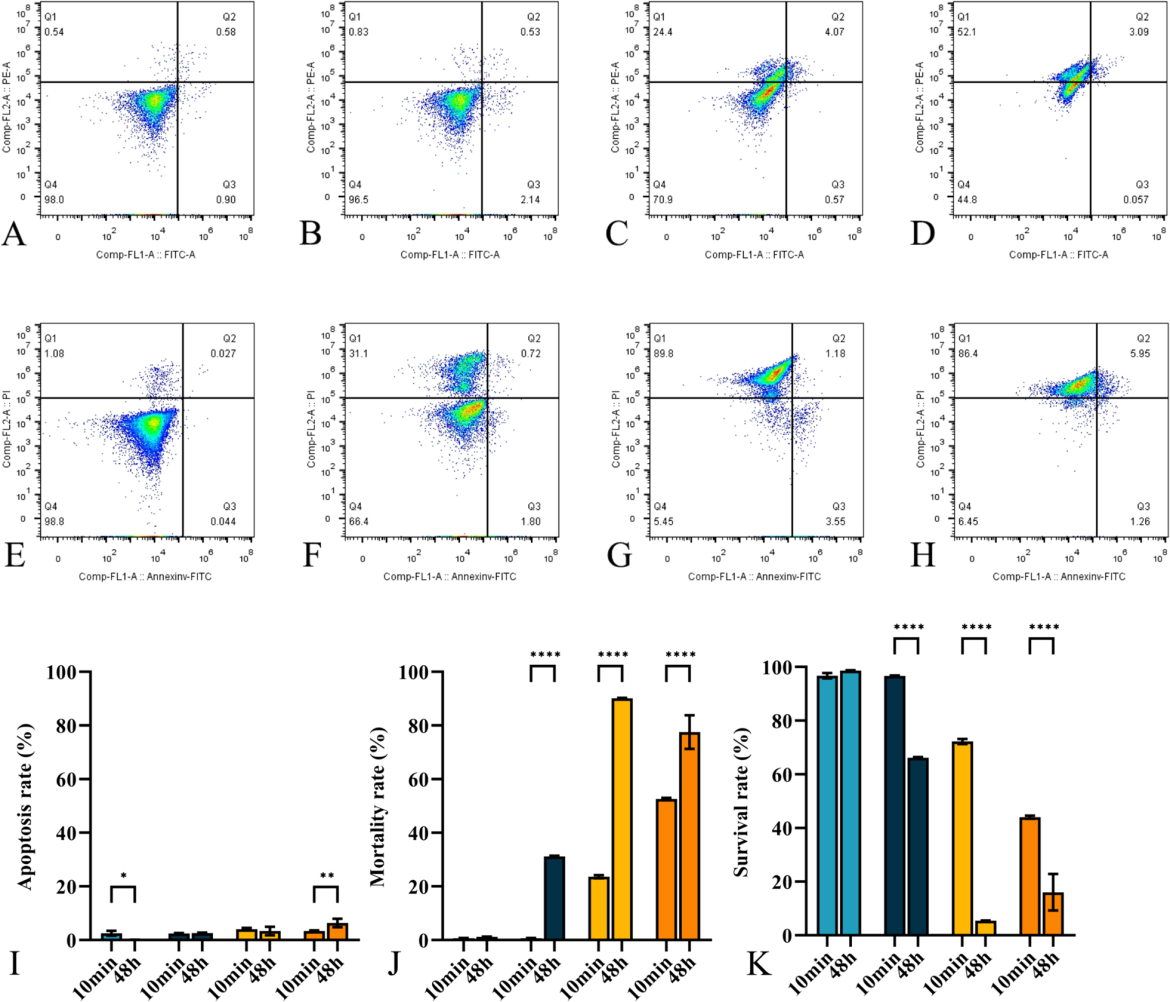
The Annexin V-FITC/PI apoptosis situation in fungi after treatment with the PS/PAPS/EC/PAEC for 10 min (**A**–**D**) and 48 h (**E**–**H**) and the apoptosis rate (**I**), mortality rate (**J**), and survival rate (**K**) in 10 min and 48 h. **A**, **E**: PS treatment, **B**, **F**: PAPS treatment, **C**, **G**: EC treatment, and **D**, **G**: PAEC treatment

Figure 4A–H illustrate the changes in apoptosis situation changes after being submerged in PS/PAPS/EC/PAEC for 10 min and 48 h. Figure 4A, B depict the fungal survival rate after 10 min of treatment with PS and PAPS, with only slight difference. However, the number of surviving fungi decreased significantly to 72.27 ± 0.97% and 44.03 ± 0.56% after immersion in EC (Fig. 4C) and PAEC (Fig. 4D), respectively. Interestingly, after 48 h immersion, the percentage of surviving fungi decreased significantly in all three groups except for PS. Specifically, the percentage of surviving fungi did not change significantly after treatment with PS (Fig. 4E), but decreased 66.20 ± 0.28% after immersion in PAPS (Fig. 4F). Notably, after 48 h of immersion in EC (Fig. 4G), the survival rate of fungi decreased more significantly than in PAEC (Fig. 4H), with percentages decreasing to 5.49 ± 0.03% and 16.08 ± 6.81% for EC and PAEC, respectively.

Figure 4I–K show the apoptosis (Fig. 4I), mortality (Fig. 4J), and survival (Fig. 4K) of the fungi after treatment with PS/PAPS/EC/PAEC for 10 min and 48 h. As shown in Fig. 4I, the increase in apoptosis rate for each group was minimal, with a maximum of no more than 7%. In contrast (Fig. 4J, K), except for PS, the mortality rate of PAPS/EC/PAEC after 48 h of immersion was much higher than after 10 min, and the survival rate was much lower. It is noteworthy that in Fig. 4 we found that PAPS had no significant inhibitory effect on fungi in a short time, but the mortality rate increased to 31.23 ± 0.25% at 48 h. This result suggests that the long-lived RS induced by plasma in PS require a longer reaction time with fungi to cause its inactivation. EC had a less inactivating effect on fungi than PAEC in a short time, but after 48 h of immersion we found that the mortality rate increased to 90.10 ± 0.22% (a rise of 66.47%) in the EC group, while the mortality rate in the PAEC group only reached 77.57 ± 6.25% (a growth of 24.97%). This means that PAEC had a significant inactivating effect in a short period of time but not as long lasting effect as EC.

### ROS detection

**Fig. 5:**
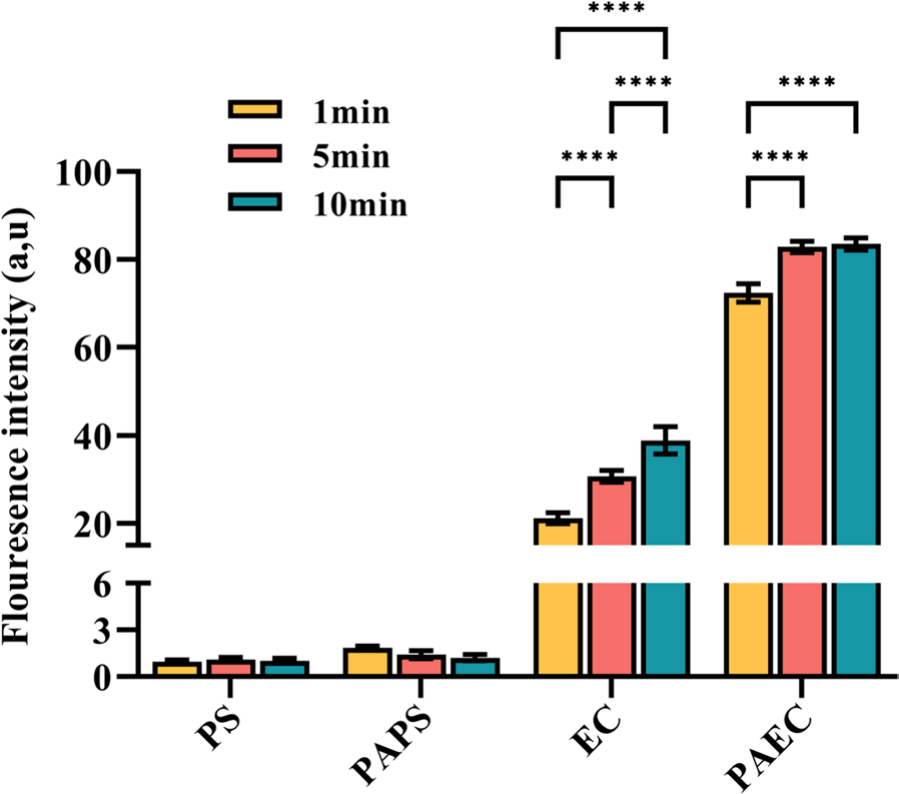
The reactive oxygen species (ROS) concentrations in fungi after treatment with the PS/PAPS/EC/PAEC

Figure 5 displays the overall intracellular ROS detection of *Candida albicans* after immersion in PS/PAPS/EC/PAEC for 1, 5, and 10 min. ROS are produced in the mitochondria as a by-product of *C. albicans* cell metabolism, providing energy for normal cellular physiological activities, and resisting oxidative stress from external damage (Langa-Lomba et al. 2021). When the level of ROS exceeds the normal metabolic range, excessive accumulation produces cytotoxicity, causing damage to intracellular lipids, DNA, proteins, and other biomolecules leading to death (Langa-Lomba et al. 2021). No essential difference was observed between fungi treated by PS and PAPS for 1, 5, and 10 min. The ROS level in the EC and PAEC groups surged rapidly and continued to grow with prolonged soaking time. It can be seen that after 10 min of treatment, the ROS concentration in the PAEC group was 2.14 times greater than that in the EC group.

### Fungal ultrastructure changes

**Fig. 6:**
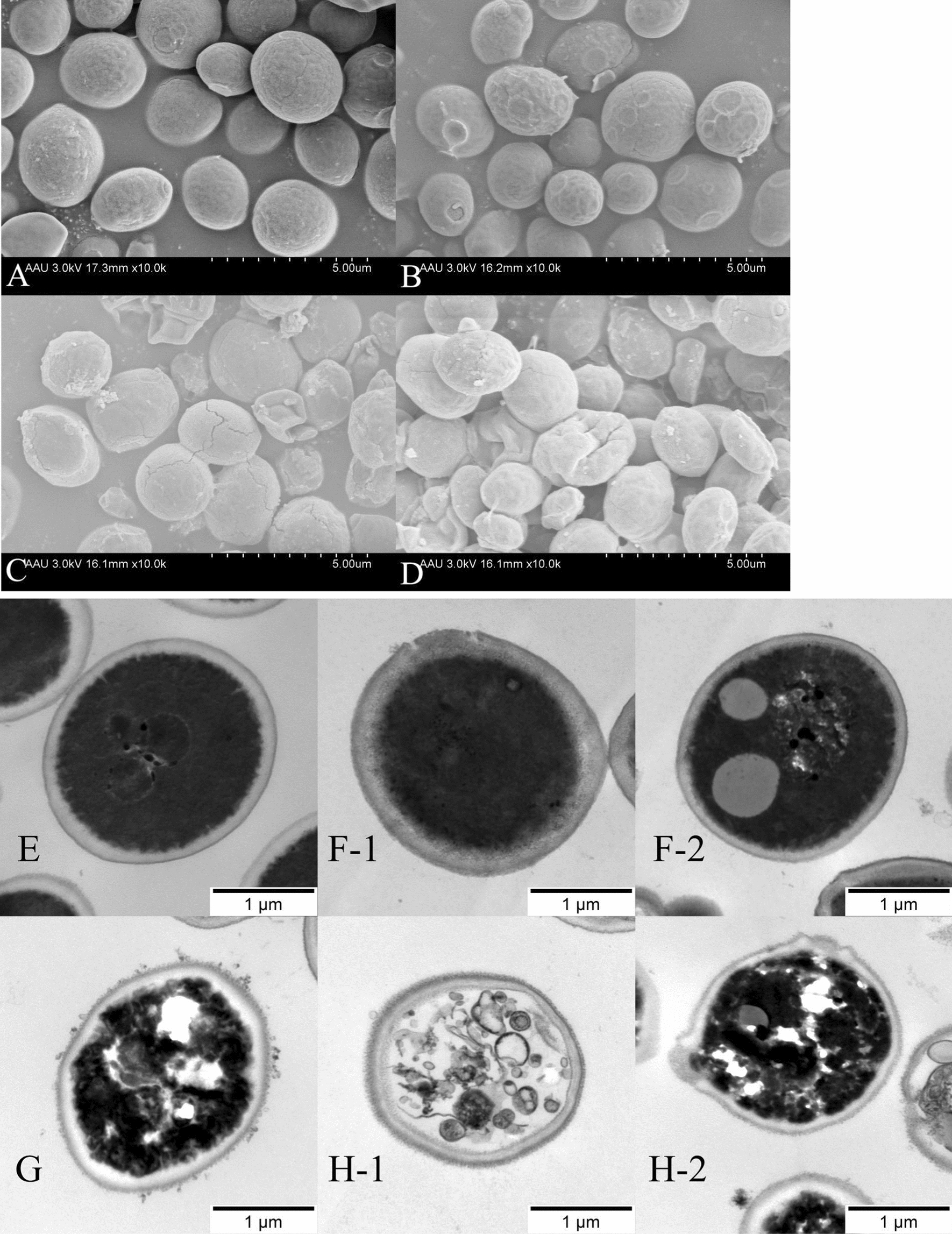
The morphological structure of *Candida albicans*were observe ed by SEM (**A**–**D**) and the micro intracellular structure of *Candida albicans*were observe ed by TEM (**E**–**H**). **A**, **E**: PS treatment, **B** F-1 and F-2: PAPS treatment, **C**, **G**: EC treatment, and **D** H-1 and H-2: PAEC treatment

SEM images showed the micromorphological changes of *C. albicans* immersed in PS/PAPS/EC/PAEC for 10 min in Fig. 6A–D. *C. albicans* cells treated with PS showed round or oval shapes, with full fungal bodies and smooth cell surfaces (Fig. 6A). Fungal cells treated with PAPS were able to maintain a better external morphology, but the surface of the fungal body began to become rough, and a few fungal bodies even ruptured (Fig. 6B). Some of the cell surfaces of *C. albicans* treated with EC were rough, wrinkled, atrophied, and the cytoplasmic contents flowed out (Fig. 6C). The majority of *C. albicans* treated with PAEC had damaged, wrinkled, and atrophied fungal cells seriously; it was difficult to find fungal cells with normal morphology (Fig. 6D).

Figure 6E–H shows TEM images of the micro intracellular changes of *C. albicans* after immersion in PS/PAPS/EC/PAEC for 10 min. *C. albicans* treated with PS had normal intact cell structure, round or oval, with smooth cell walls and intact, clear cell membranes (Fig. 6E). The damage to the organism treated with PAPS was relatively small, with normal cells predominating; the cell wall of damaged cells became thicker, its integrity was destroyed, and the cell membrane was discontinuous (Fig. 6F-1); or the cytoplasm had edema, shallow density, nuclear material aggregated at the edge, and vacuolation occurred (Fig. 6F-2). The damage to fungal bodies treated with EC was heavier, with abnormal fungi predominating; the cell wall of damaged cells was damaged, showing distorted and irregular shapes, with vacuole formation in the cytoplasm (Fig. 6G). The damage to fungi treated with PAEC was heavier, with almost no normal fungal structure; the cell wall of damaged cells was damaged, showing distorted and irregular shapes, the cell membrane was destroyed, and part of the cytoplasm flowed out of the cell wall (Fig. 6H-1); or the cytoplasm even appeared vacuolated structure cell nucleus rupture, organelle dissolution, cytoplasm into crumb-like (Fig. 6H-2).

### Virulence factors

#### Adhesion to tissue surfaces impacts and CSH effects

**Fig. 7:**
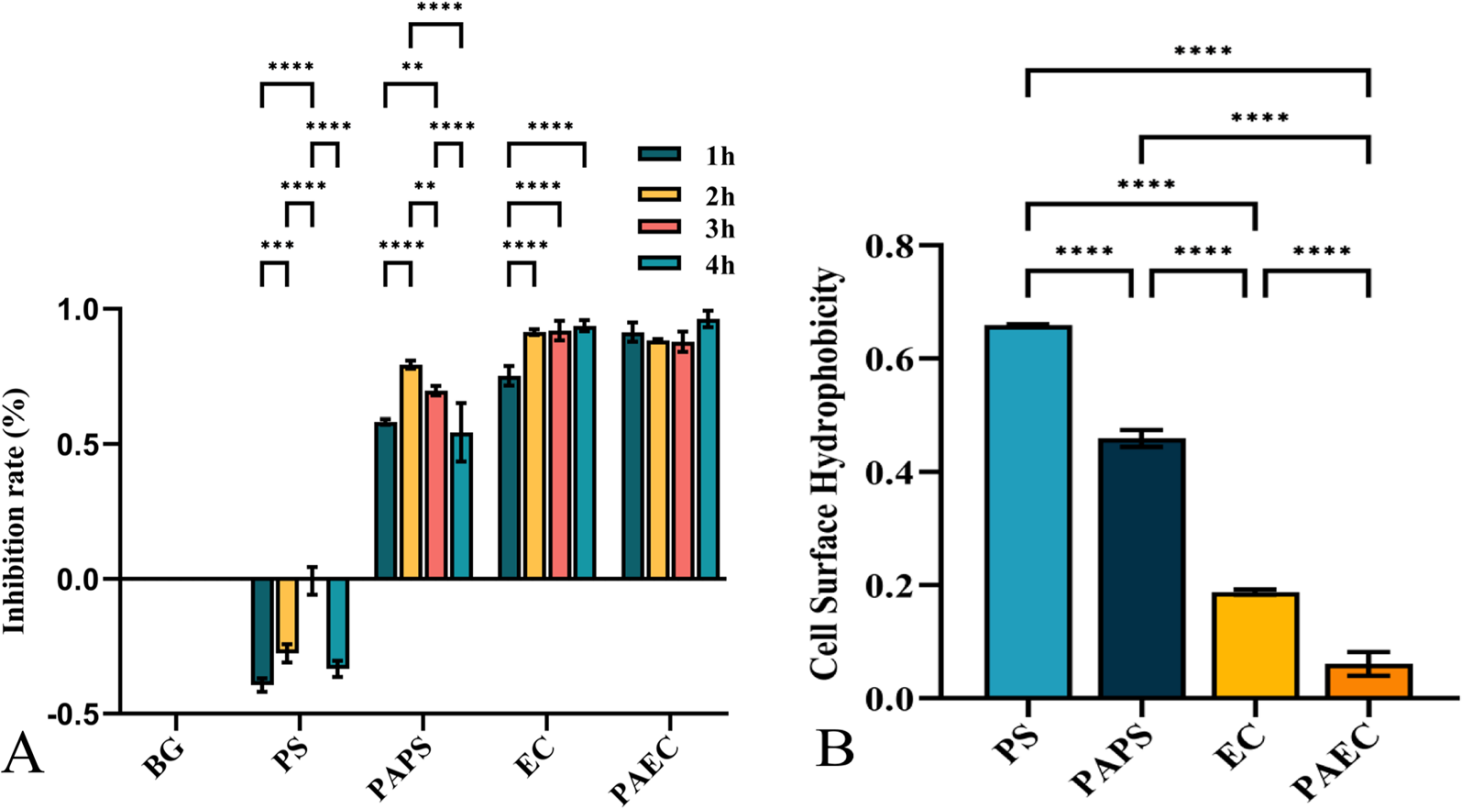
Adhesion to tissue surfaces impact (**A**) and CSH effects (**B**) of fungi after treatment with the PS/PAPS/EC/PAEC

Figure 7A shows the adhesion to tissue surfaces impacts the fungal biofilms were incubated for 1, 2, 3, and 4 h and then immersed in PS/PAPS/EC/PAEC for 24 h. Strong CSH and adhesion to inanimate material surfaces are characteristics of *C. albicans* with strong virulence factors. As a result, these fungi are very likely to adhere to implants and interventional catheters, allowing for quick multiplication on the surface of the host and the development of invasive fungal infections (Suchodolski et al. 2020). The adhesion rate of early biofilms (1, 2, 3, 4 h) of *C. albicans* after PS immersion was promoted compared to the blank group (BG). In contrast, PAPS inhibited the adhesion rate of early biofilms, and the inhibition was greatest at 2 h of biofilm growth, with a peak value of 0.793 ± 0.012, and then the inhibition of PAPS decreased with the extension of biofilm incubation time. Likewise, the adhesion rate of biofilms incubated for 1 h was inhibited by EC up to 0.752 ± 0.029, and the inhibitory effect grew stronger over time before gradually stabilizing. The inhibition rate of PAEC on biofilms incubated for 1 h was 0.914 ± 0.029, and it did not change significantly with the increase of incubation time on biofilms. Figure 7B shows the CSH effects of the fungal biofilm after being submerged in PS/PAPS/EC/PAEC for 24 h. The surface hydrophobicity of *C. albicans* immersed in PAPS decreased by 30.56% compared to PS, reaching 0.459 ± 0.012. The CSH of both EC/PAEC immersion decreased more substantially, and PAEC has more effective inhibition on the CSH of *C. albicans*. In this study, we found that both PAPS/EC/PAEC reduced the surface hydrophobicity of *Candida*, which in turn inhibited the adhesion of *Candida* to tissues and inhibited the formation of early biofilm.

#### Yeast-phase cells to mycelium-phase cells transform

**Fig. 8:**
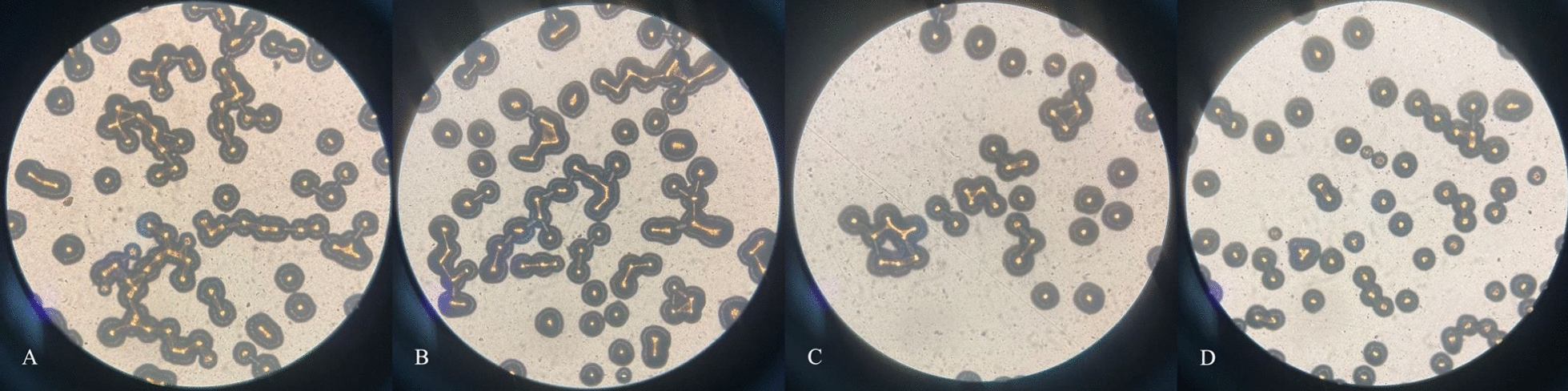
Fungal structures were photographed under an optical microscope. (**A**–**D**): **A**: PS treatment, **B**: PAPS treatment, **C**: EC treatment, and **D**: PAEC treatment

Figure 8A–D shows the conversion of yeast-phase cells to mycelium-phase cells following a 10 min immersion in PS/PAPS/EC/PAEC and a 24 h incubation period on Spider agar plates. The morphological transformation of *C. albicans*, in which the yeast phase is linked to attachment and dissemination while the mycelial phase enables *Candida* to be more invasive, invade adherent host tissues more effectively, and form mature biofilms that evade host phagocytosis, is crucial to the pathogenesis of host infection (Bonhomme et al. 2013). *C. albicans* treated with PS and PAPS showed a large number of yeast-phase cells converting to mycelium-phase cells, with intertwined mycelium and high density (Fig. 8A, B); EC treatment showed almost all yeast-phase and a small amount of mycelium-phase with low density (Fig. 8C), and PAEC treatment showed only yeast-phase cells with low density (Fig. 8D). These findings demonstrate that, in comparison to PS and PAPS, EC and PAEC were able to inhibit mycelium formation and thus biofilm production.

#### Secretion of hydrolytic enzymes activities

**Fig. 9:**
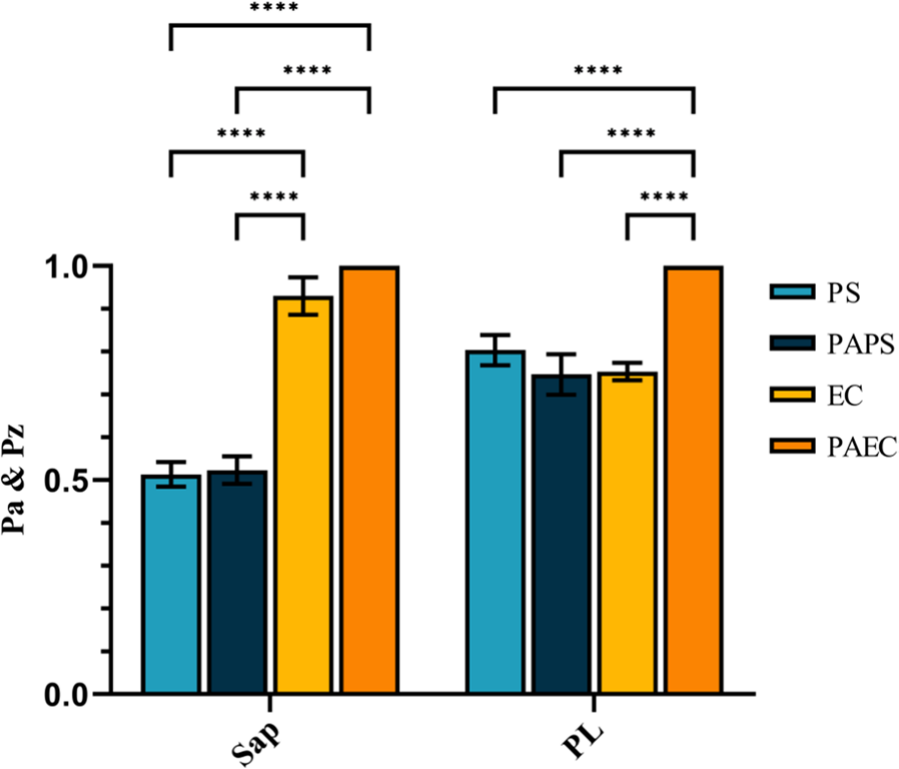
Secretion of hydrolytic enzymes (Sap and PL) activities of fungi after treatment with the PS/PAPS/EC/PAEC

Figure 9 shows the Sap and PL activities that were secreted after 10 min immersion in PS/PAPS/EC/PAEC. Sap and PL are significant virulence factors of *C. albicans* that can hydrolyze host cell proteins and phospholipids, respectively. They also play a critical role in evading the host immune system and the action of antifungal drugs, making clinical treatment challenging (Gropp et al. 2009). PL and Sap are regarded as two of the most significant virulence factors. In our study, neither Sap nor PL colonies were observed in the area where *C. albicans* were soaked in PAEC for 10 min before being cultured for 48 h. (According to the formula, both Pa and Pz values are 1). For Sap, the Pa values of fungi treated with EC shows a significant increase at 0.93 ± 0.039, and there was no difference between fungi treated with PS and PAPS. For phospholipase, there was essentially no variation in the Pz values of fungi treated with PS, PAPS, and EC. According to the findings, PS, PAPS, and EC, with the exception of PAEC, had no discernible inhibitory effect on the secretion of PL. Additionally, when compared to PS and PAPS, EC and PAEC significantly inhibited Sap secretion.

## Discussion

After plasma treatment of the liquid, liquid-phase RS is produced, including long-lived H_2_O_2_, H^−^, ${\text{NO}}_{3}^{ - }$, and O_3_ and short-lived^.^OH, O^−^, NO, ONOO^−^, and ClO_3_ (Naïtali et al. 2010; Brisset et al. 2011). In this study, plasma-activated TCM solution was used, and the liquid-phase short-lived RS produced by the direct action of the plasma dissipate shortly after the plasma is turned off, so they are not the main antifungal factor. Therefore, we focus on the long-lived RS of the plasma-activated solution, such as H_2_O_2_, ${\text{NO}}_{3}^{ - }$, and O_3_, in this investigation. The results showed that the concentrations of H_2_O_2_, ${\text{NO}}_{3}^{ - }$ and O_3_ increased after plasma treatment (Fig. 2A) and the pH was significantly reduced (Fig. 2B).

When PS is exposed to plasma, H_2_O is directly decomposed (Xu et al. 2018b):

1 $${\text{H}}_{{2}} {\text{O}} + {\text{ e}}^{ - } \to^{.} {\text{H}} +^{.} {\text{O}} + {\text{ e}}^{ - }.$$

2 $${\text{H}}_{{2}} {\text{O}} + {\text{ e}}^{ - } \to {\text{H}}_{{2}} {\text{O}}^{ + } + {\text{ 2e}}^{ - }.$$

3 $${\text{H}}_{{2}} {\text{O}}^{ + } + {\text{ H}}_{{2}} {\text{O}} \to^{.} {\text{OH}} + {\text{ H}}_{{3}} {\text{O}}^{ + }.$$

In the case of air as a carrier gas, the gas phase of nitrogen and oxygen can be dissociated and form nitrogen oxides (Zhang et al. 2017):

4 $${\text{O}}_{{2}} + {\text{ e}}^{ - } \to {\text{2O}} + {\text{ e}}^{ - }.$$

5 $${\text{N}}_{{2}} + {\text{ e}}^{ - } \to {\text{2N}} + {\text{ e}}^{ - }.$$

6 $${\text{N}} + {\text{ O}} \to {\text{NO}}.$$

7 $${\text{N2}} + {\text{ O}} \to {\text{N}} + {\text{ NO}}.$$

8 $${\text{NO}} + {\text{ O}} \to {\text{NO}}_{{2}}.$$

9 $${\text{NO}} + {\text{ O}}_{{3}} \to {\text{NO}}_{{2}} + {\text{ O}}_{{2}}.$$

Atomic oxygen combines with O_2_ to form O_3_ or reacts with H_2_O to form hydroxide radicals and hydrogen peroxide in liquids (Du et al. 2008; Burlica et al. 2006):

10 $${\text{O}} + {\text{ O}}_{{2}} \to {\text{O}}_{{3}}.$$

11 $${\text{OH}} +^{.} {\text{OH}} \to {\text{H}}_{{2}} {\text{O}}_{{2}}.$$

12 $${\text{O}} + {\text{H}}_{{2}} {\text{O}} \to {\text{H}}_{{2}} {\text{O}}_{{2}}.$$

13 $${\text{2H}}_{{2}} {\text{O}} + {\text{ e}}^{ - } \to {\text{H}}_{{2}} + {\text{ H}}_{{2}} {\text{O}}_{{2}} + {\text{ e}}^{ - }.$$

High concentrations of H_2_O_2_ are associated with DNA, lipid, and protein damage (Xu et al. 2018). When H_2_O_2_ is elevated to a certain level, oxidative stress is generated in cells, causing an increase in intracellular ROS in mycelium, leading to a decrease in total peroxidase activity and peroxidase gene expression, which inhibits mycelium formation and ultimately reduces the pathogenicity of the bacterium (Yang et al. 2017).

The formation of nitrite is based on the following reactions (Zhang et al. 2017):

14 $${\text{NO}} + {\text{ NO}}_{{2}} + {\text{ H}}_{{2}} {\text{O}} \to {\text{2HNO}}_{{2}}.$$

15 $${\text{NO}} +^{.} {\text{HO}}_{{2}} \to {\text{HNO}}_{{2}}.$$

In microbial systems, nitrite reacts with metal ions present in enzymes leading to severe nitrosative stress and impeding normal ATP oxidative metabolic processes (Tharmalingam et al. 2017). In this study, nitrite was not detected since it is easily converted to nitrate in acidic conditions and also reacts with hydrogen peroxide to form peroxynitrite (Lukes et al. 2014). Peroxynitrite is a strong oxidant that reacts with most biological molecules, leading to protein alterations and promoting cellular damage through direct reaction with proteins such as tyrosine and cysteine (Tharmalingam et al. 2017).

16 $${\text{NO}}_{{2}} + \, \cdot {\text{OH}} \to {\text{HNO}}_{{3}}.$$

17 $${\text{2NO}} + {\text{ H}}_{{2}} {\text{O}} + {\text{ O}}_{{3}} \to {\text{2HNO}}_{{3}}.$$

18 $${\text{NO}}_{{2}}^{ - } + {\text{ H}}_{{2}} {\text{O}}_{{2}} + {\text{ H}}^{ + } \to {\text{ONOOH}} + {\text{ H}}_{{2}} {\text{O}}.$$

The combination of ONOOH in an acidic environment greatly contributes to the bactericidal effect of the plasma treatment solution (Oehmigen et al. 2011), and ONOOH eventually decomposes to ${\text{NO}}_{3}^{ - }$ (Tharmalingam et al. 2017).

19 $$\,{\text{ONOOH}} \to {\text{NO}}_{{2}} + \, \cdot {\text{OH}}.$$

20 $${\text{ONOOH}} \to {\text{HNO}}_{{3}} \to {\text{NO}}_{{3}}^{ - } + {\text{ H}}^{ + }.$$

In this study, there was no significant inactivation effect from PAPS for a short period of time. This may be because H_2_O_2_ and O_3_ had less impact on the fungus, and secondly, ONOOH, the secondary product had a small effect on the fungus. Alternatively, because nitrite was not measured, this reaction may not have occurred (18), and there was no ONOOH. Additionally, we discovered that PAPS did not have a major inhibitory effect on the fungus in a short amount of time, but it did have an inactivating effect for a long enough period of time, just not as strong as the TCM solution. This could be due to the fungal cell wall (composed of 90% cellulose and 10% protein) preventing RS in PAPS from harming cells, but if the immersion time is long enough, RS can also harm the protein components of the cell wall, lysing it and causing cell contents to leak out, ultimately inactivating *Candida albicans*. Our conjecture was indirectly confirmed by a study that the number of culturable *Aspergillus flavus* spores was significantly reduced after a period of immersion due to the synergistic effect between acidification of plasma-activated water (PAW) and long-lived RS leading to fungal cell wall disruption (Los et al. 2020).

The analysis of PAEC showed that the concentrations of oxymatrine and rhein, the drug representative components, were both increased after plasma treatment (Fig. 2C). Because both EC and PAEC were brownish yellow, the RS content could not be detected by the H_2_O_2_, ${\text{NO}}_{3}^{ - }$ and O_3_ kit, but by the detection of PAPS, we inferred that RS would be produced in the EC after plasma treatment. Oxymatrine, the active ingredient of the Chinese herb bitter ginseng, is an alkaloid with inhibitory or inactivating effects on both *Candida albicans* and *Candida* biofilms (Wu et al. 2009; Shao et al. 2014). Rhein, a common organic acid, has a beneficial effect on *C*. *albicans*, *Trichoderma*, and *Aspergillus fumigatus* with antifungal activity (Agarwal et al. 2000). The HPLC results (as shown in Fig. 2C) showed that the concentrations of these two active components increased significantly after plasma treatment. The reasons for this are not clear, but we speculate on the following possibilities. One is that after plasma treatment, the volume of liquid decreases by about 50% due to evaporation. Another is that after plasma treatment, the pH value was reduced and the acid could promote the leaching of alkaloids and also the freeing of organic acids, increasing the solubility of alkaloids and organic acids (Ribeiro et al. 2008). As to how much or whether these factors are the reasons for concentrations of herb increasing in this study needs to be further investigated subsequently.

Various natural compounds in Chinese medicine can exert their anti-*Candida* activity through different mechanisms such as cell membrane, cell wall, mitochondria, and virulence factors (Sarangapani et al. 2019). In clinical practice, a combination of two or more drugs is often required to control *Candida albicans* infections in vivo (Chang et al. 2012). Plasma inhibited *S. aureus*, *S. epidermidis*, *Pseudomonas aeruginosa*, *Escherichia coli* and *Candida albicans* in vitro, but plasma was the least effective against *C. albicans*. Through our study, it was found that PAW also failed to provide significant inhibition against *C. albicans*, but PAEC could improve this problem well. By CFU results (Fig. 3A), we observed that the number of *C. albicans* soaked in both EC and PAEC had a decreasing trend and was time-dependent. The inactivation number was basically the same when EC and PAEC were soaked for 0–1 min, while PAEC decreased significantly compared to EC after 1 min, indicating that there were other antifungal components in PAEC in addition to herbal components. The additional antifungal properties of PAEC may come from two reasons. One is that RS induced by plasma in the liquid have no significant antibacterial effect on *C. albicans* alone, but when RS are combined with herbal components, they may be more antibacterial than one substance (such as liquid RS or herbal components) alone. Although there is no relevant literature on this synergistic effect of Chinese herbal medicine and liquid-phase RS, this inference can be indirectly confirmed based on our experimental results. Another reason is that plasma can degrade organic matter in water (Brisset et al. 2011). As an effective component of traditional Chinese medicine, it can be regarded as a kind of organic matter dissolved in liquid, which can be oxidized and decomposed by plasma acting on the liquid. This decomposition process may produce stronger chemical components that inactivate *C. albicans*, which are similar to plasma degrading organic pollutants in water to form more toxic small molecule components. Whether this factor causes PAEC to have a stronger antifungal effect in a short period of time needs further study.

*C. albicans* is a fungus with a cell wall, which is a rigid structure located in the outermost layer of the cell that maintains cell shape and protects the cell from external factors through this barrier and is the first line of defense of the cell (Chaffin 2008). The cell membrane, located on the inner side of the cell wall, is the second barrier of the fungal cell and an important structure for maintaining cell shape as well as regulating the virulence of *Candida* (Zorić et al. 2017). When a significant increase in AKP or protein molecules is measured, it indicates that the integrity of the *Candida* cell wall or cell membrane is damaged (Fig. 3B, C). When fungal cell membranes are damaged by antifungal drugs, the permeability of the membranes is altered so that intracellular substances leak out and antifungal drugs thus exert their antifungal effects intracellularly, affecting various physiological metabolic activities of cells and eventually leading to the death of the fungal cells (Corrêa et al. 2020).

We used Annexin V-FITC and PI (AV/PI) labeling tests to determine whether the death of *Candida* cells induced by PAPS, EC, and PAEC were completed by the necrotic or apoptotic pathway. Through AV/PI experiments (Fig. 4A–H), we discovered that PAPS could not completely inhibit the growth of the fungus and did not have an inactivating effect by short immersion. However, an increase in the number of specific necrosis could be observed at 48 h, proving that PAPS also had a destructive effect on the structure of the fungus. *Saccharomyces cerevisiae*, like *C. albicans*, have a eukaryotic cell wall, and damage to the cell wall in combination with PAW and sodium laureth sulfate (SLES) leads to leakage of proteins and nucleic acids, while RS in PAW induces severe oxidative damage to other cellular components such as thiols, DNA and proteins, which may eventually lead to cellular necrosis (Liu et al. 2021b). Additionally, Fig. 3 revealed that the inhibition of *Candida* by EC and PAEC both had the impact of damaging the cell structure, but it is worth noting that the sterilizing impact of PAEC is superior in a short amount of time, whereas EC has a better and longer sterilization effect for 48 h in this research. Therefore, we hypothesize that the long-lived RS in PAPS contributes to the inhibition process, whereas in PAEC, it is the reaction of Chinese medicine ingredients with plasma-induced liquid-phase RS that breaks down into small-molecule substances with enhanced ability to destroy fungal cell structure in a short amount of time, though such small-molecule substances may be unstable and time-limited. As a result, the inhibitory effect of PAEC acting on *Candida albicans* for a short period of time and a long period of time differs.

In this study, it was discovered that both EC and PAEC caused *C. albicans* to accumulate intracellular ROS within a short amount of time, which ultimately resulted in fungal mortality because of intracellular ROS buildup (Fig. 5). The cause may be connected to the fact that herbal ingredients have been shown to degrade DNA, upregulate the expression of mycelium-specific genes, and downregulate the key morphogenetic regulator Ras1p and intracellular ROS levels (Guirao-Abad et al. 2017). After plasma treatment, RS in PAEC can cross the membrane to oxidize DNA or cause peroxidation of reactive oxygenated lipids, with the end product triggering oxidation and leading to subsequent DNA damage (Kim et al. 2021). The excessive fluorescence intensity of intracellular ROS after EC and PAEC treatment may be partly caused by the color of EC or PAEC, in addition to the increase of intracellular ROS caused by EC and PAEC treatment.

Using SEM and TEM to examine the morphology and ultrastructure of *Candida* (Fig. 6A–H), it was discovered that PAPS, EC, and PAEC could alter the morphological structure of *C. albicans* cells. Compared to PS immersion, we discovered that the cytoplasm of the fungus was lysed after EC immersion, and the morphology of necrotic fungal cells following PAPS and PAEC immersion were essentially classified into two types: cell wall rupture and cytoplasm lysis. The oxymatrine and rhein in EC were shown to destroy the normal cell structure of the fungus, leading to leakage of cell contents and eventual inactivation (Shao et al. 2014; Agarwal et al. 2000). There are two more mechanisms for PAPS and PAEC to destroy fungus cells. On the one hand, after RS reacts with the cell for a long enough period of time, the cell wall and cell membrane are broken, resulting in the release of cell contents. On the other hand, it might result from RS entering the inside of fungal cells from PAPS and PAEC and reacting with the macromolecules (proteins, nucleic acids, etc.) Based on the results of more severe necrosis of the fungus after PAEC immersion, we speculated that the TCM solution after plasma treatment not only inherited the anti-fungal effects of TCM and RS, but also produced antifungal substances with stronger effects other than the two themselves, or the two exerted a synergistic effect to destroy the fungal structure.

Due to its tremendous adaptability and ability to transform from a commensal to a pathogenic state through various virulence factors, *C. albicans* can attach to an host and cause a wide range of diseases, from cutaneous mucosal infections to systemic infections. In Particular, the ability to alter morphology and form biofilms is essential to the pathogenicity of *Candida albicans* (Staniszewska et al. 2012). Additionally, *C. albicans* is more resistant to traditional antifungal medications due to its ability to form biofilms. Successful colonization and persistence during infection require *Candida* to be able to attach to the host surface (Rahimi-Verki et al. 2016). As shown by the CCK-8 experiment, the *Candida albicans* biofilm in PS/PAPS/EC/PAEC immersion had an impact on the surface adhesion of the fungus. It's interesting that PAPS, EC, and PAEC all inhibited surface adhesion on the metabolic activity of *Candida*, while PS had the opposite effect (Fig. 7A). The inhibitory effect of PAPS on biofilm during the beginning growth peaked at 2 h, indicating that PAPS had some inhibitory effect on biofilm in the early stage of growth; however, as the biofilm developed, RS was unable to penetrate it, and the inhibitory effect steadily reduced.

We discovered that the CSH of *Candida* cultured in PAPS/EC/PAEC was lower than that after PS treatment using the water-hydrocarbon two-phase assay (Fig. 7B). CSH, an intrinsic property of the outer cell wall layer, allows *Candida albicans* to bind strongly and form aggregates. This force maintains the optimal distance between adhesion molecules and host receptors for strong binding and ultimately irreversible adhesion to mucous membranes or other substrates. CSH may affect biofilm formation as adhesion is a crucial first step in biofilm development (Zupancic et al. 2020; Muadcheingka et al. 2015).

In their yeast state, *Candida* cells attach to the host surface, generate a significant amount of hyphae, and secrete an extracellular matrix. Together, these actions create a dense, reticular mature biofilm structure (Chen et al. 2020). Current medications for treating *Candida* biofilms have limited sensitivity (Mathé et al. 2013). The production of mature biofilms in *Candida* increases resistance to antifungal treatments and correlates with higher mortality rates in patients with candidiasis. In this study, we used light microscopy (Fig. 8) to observe that PS and PAPS had little to no inhibitory effect on mycelium growth. In contrast, EC and PAEC prevented mycelium growth, reduced the capacity to form budding tubes, inhibited morphological changes in *C. albicans* from changing its morphology, and effectively prevent the formation of biofilm. Our results are similar to a previous study that reported berberine hydrochloride disrupts biofilm structure and inhibits *Candida albicans* biofilm formation by down-regulating the expression of *EFG 1*, *HWP 1*, *ECE 1* and *ALS 1* during mycelium formation, thereby delaying the morphological transformation of the mycelium (Huang et al. 2020).

In addition to mediating the adhesion and invasion of the fungus to host cells and tissues, Sap has a high protein hydrolase activity and can hydrolyze a wide range of host substrates (Stehr et al. 2000). This provides nutrients for *C. albicans* growth by destroying various protective molecules (such as mucins) on the mucosal surface, while also enhancing its adhesion and invasion ability and fostering the invasion of host tissues (Villar et al. 2007). Our results indicate that RS in PAPS could not prevent *C. albicans* from secreting Sap, as PAPS had minimal effect on reducing Sap production compared to PS (Fig. 9). Contrarily, EC effectively reduced Sap secretion, attenuating host cell protein hydrolysis, and preventing adherence and invasion of the organism to host cells and tissues. No colonies were observed on bovine serum albumin agar plates, indicating that PAEC inhibited Sap secretion. The pathogenic mechanism of PL may also involve direct host cell damage and cell hydrolysis (Ghannoum 2000). Numerous secretory PL contain glycosylated moieties that facilitate cell membrane and/or cell wall binding and secretion. These properties enable *Candida albicans* to invade, survive, and be pathogenic in vivo, as well as evade host immune response mechanisms (Djordjevic 2010; Naglik et al. 2003). According to the results (Fig. 9), neither PAPS nor EC significantly decreased PL secretion compared to PS, indicating that their inactivation effect may not be related to a decrease in virulence factors. Furthermore, no colonies were observed on yolk agar plates, indicating that PAEC inhibited PL secretion. Based on our findings, we suggest that PAEC may have some inhibitory effect on the secretion of both Sap and PL.

PAW can serve as a novel disinfectant solution for skin protection, producing RS such as H_2_O_2_ and O_3_ that are non-toxic to normal skin cells and exhibit strong bactericidal effects (Lee et al. 2022). Plasma-activated hydrogels have been shown to effectively and safely treat skin lesions in vitiligo patients, with no adverse events observed in clinical trials or during follow-up (Zhai et al.^2021^). In one study, immunodeficient nude mice were given PAW orally, and following treatment, there were no appreciable alterations in their blood biochemical parameters or tissue structure, proving that PAW had no substantial safety issues (Xu et al. 2018a). In recent years, external therapy using TCM has demonstrated positive results in treating diabetic peripheral vascular disease, psoriasis and other skin diseases without adverse effects, confirming the cellular safety of aqueous solutions of Chinese herbs (Ding et al. 2022; Zhang et al. 2022). Nowadays, despite the fact that many individuals investigate plasma-activated liquids, the majority of studies tend to focus on their sterilisation or degrading processes rather than their safety. Our study is the first to combine TCM with plasma, and only focus the enhancement of antifungal effects of plasma-activated TCM. We might need to do study on the PAEC-related biosafety in the future.

**Fig. 10:**
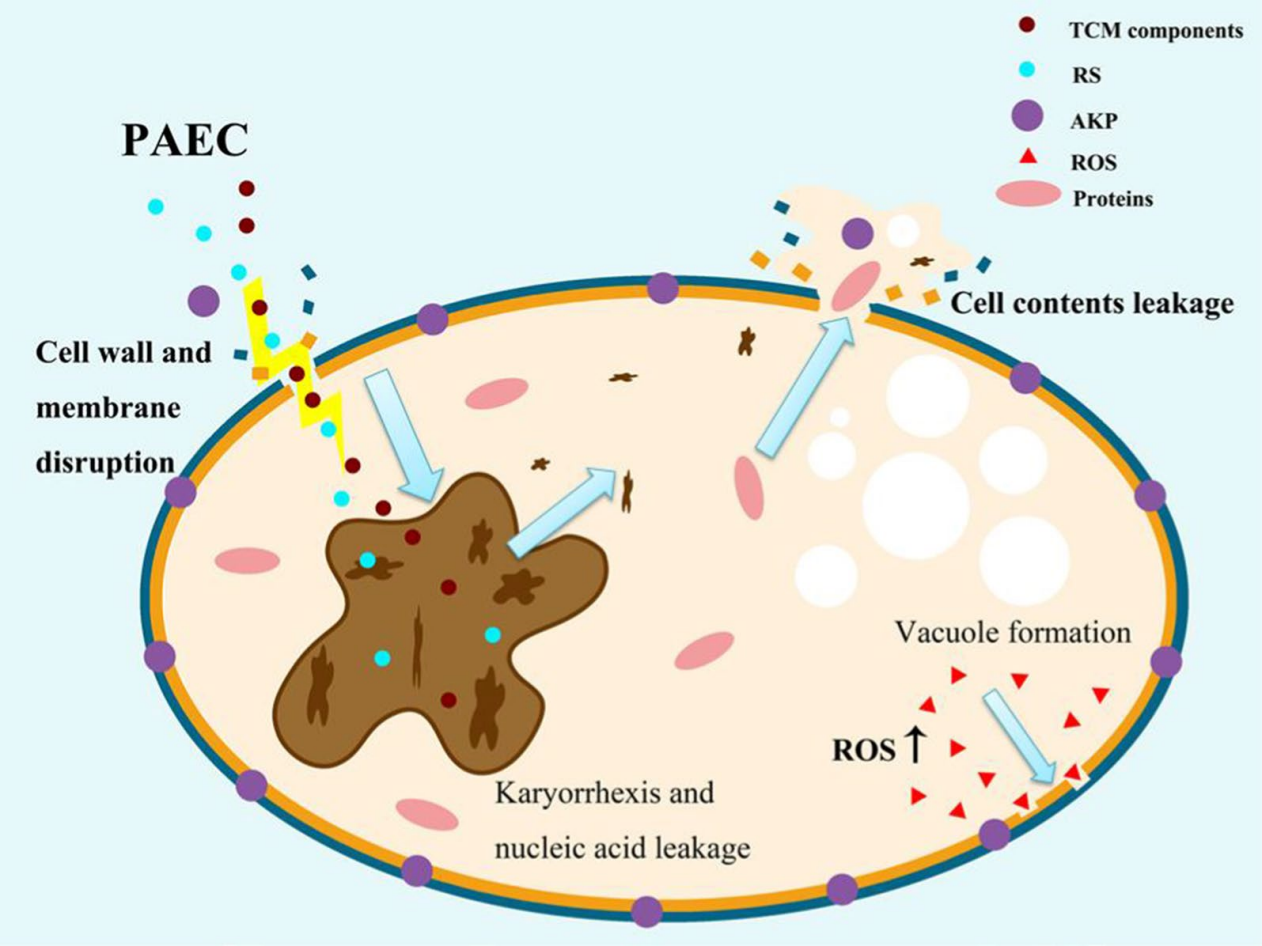
Mechanism of the synergistic antifungal effect by RS and TCM components in PAEC

Our findings indicate that plasma-initiated liquid-phase long-lived RS were less destructive to *Candida albicans* cell walls and membranes than TCM components in EC. The mechanism of *Candida albicans* inhibition by the synergistic effect of RS and TCM components in PAEC is depicted in Fig. 10, and primarily includes the following interactions and pathways: (1) TCM components and RS in PAEC, or new components induced by plasma in PAEC, alter the morphology of *C. albicans* cells, cuasing cell wall and membrane rupture, leakage of intracellular material, and even leading to the occurrence of karyorrhexis and nucleic acid exudation; (2) TCM components and RS in PAEC enter fungal cells and trigger oxidative stress in through oxidation, leading to a substantial increase in intracellular ROS content and ultimately causing cell necrosis.

In summary, we systematically investigated the antifungal ability of PAEC on *C. albicans*. According to HPLC results, the oxymatrine and rhein concentrations increased in PAEC compared to EC. Plasma treatment elevated RS levels and lowered pH in PS. The CFU test showed reduction of approximately 3 orders of magnitude in fungal colonies after 10 min of PAEC. Furthermore, PAEC immersion induced morphological changes in the fungus, including disruption of cell walls and membranes, leakage of intracellular material, and disruption of ROS balance. In addition, PAEC immersion reduced virulence factors, including reduction of adhesion to tissue surfaces and CSH, the transformation of yeast-phase cells to mycelium-phase cells, and the decrease in the secretion of hydrolytic enzymes.

## Data Availability

The datasets generated during and analysed during the current study are available from the corresponding author on reasonable request.
